# Identification of Cyanobacteria and Its Potential Toxins in the Joanes I Reservoir, Bahia, Brazil

**DOI:** 10.3390/toxins15010051

**Published:** 2023-01-06

**Authors:** Maria Teresa Araujo Pinheiro Menescal, Edna dos Santos Almeida, Emerson Andrade Sales, Annick Méjean, Claude Yéprémian

**Affiliations:** 1 Laboratory of Bioenergy and Catalysis (LABEC), Polytechnic School, Federal University of Bahia—UFBA, Rua Aristides Novis, 2, 2nd Floor, Federação, Salvador 40210-910, BA, Brazil; 2 Industrial Engineering Post-Graduation Program (PEI), Polytechnic School, Federal University of Bahia—UFBA, Rua Aristides Novis, 2, 6th Floor, Federação, Salvador 40210-910, BA, Brazil; 3 Senai Cimatec University Center, Orlando Gomes Avenue, Salvador 41650-010, BA, Brazil; 4 LIED, UMR 8236 CNRS, Université Paris Cité, 75205 Paris, France; 5 UMR 7245 Molécules de Communication et Adaptations des Microorganismes (MCAM), Muséum National d’Histoire Naturelle, CNRS, CP 39, 57 rue Cuvier, 75005 Paris, France

**Keywords:** environmental monitoring, cyanotoxins, molecular and chemical detection, harmful cyanobacteria blooms, drinking water

## Abstract

The Joanes I Reservoir is responsible for 40% of the drinking water supply of the Metropolitan Region of Salvador, Bahia, Brazil. For water sources such as this, there is concern regarding the proliferation of potentially toxin-producing cyanobacteria, which can cause environmental and public health impacts. To evaluate the presence of cyanobacteria and their cyanotoxins in the water of this reservoir, the cyanobacteria were identified by microscopy; the presence of the genes of the cyanotoxin-producing cyanobacteria was detected by molecular methods (polymerase chain reaction (PCR)/sequencing); and the presence of toxins was determined by liquid chromatography with tandem mass spectrometry (LC-MS/MS). The water samples were collected at four sampling points in the Joanes I Reservoir in a monitoring campaign conducted during the occurrence of phytoplankton blooms, and the water quality parameters were also analysed. Ten cyanobacteria species/genera were identified at the monitoring sites, including five potentially cyanotoxin-producing species, such as *Cylindrospermopsis raciborskii*, *Cylindrospermopsis* cf. *acuminato-crispa*, *Aphanocapsa* sp., *Phormidium* sp., and *Pseudanabaena* sp. A positive result for the presence of the cylindrospermopsin toxin was confirmed at two sampling points by LC-MS/MS, which indicated that the populations are actively producing toxins. The analysis of the PCR products using the HEPF/HEPR primer pair for the detection of the microcystin biosynthesis gene *mcy*E was positive for the analysed samples. The results of this study point to the worrisome condition of this reservoir, from which water is collected for public supply, and indicate the importance of the joint use of different methods for the analysis of cyanobacteria and their toxins in reservoir monitoring.

## 1. Introduction

Cyanobacteria are phytoplankton microorganisms, known as blue-green algae, and are found in lakes and surface water reservoirs. There is a wide variety of species that produce toxic compounds and that can proliferate excessively, forming blooms under the favourable environmental conditions of temperature, the presence of nutrients, and light [1,2].

Therefore, the occurrence of these species constitutes a major threat to public health and aquatic ecosystems, and incidents of animal and human poisoning, as well as the disease-causing effects due to toxic cyanobacterial blooms, are of concern all over the world [3,4,5].

There have been reports of phytoplankton blooms with the presence of cyanobacteria in Brazilian reservoirs, mainly in the Northeast Region [6], which is worrisome because several cyanobacterial species produce metabolites which are generically called cyanotoxins [7]. The presence of these toxins can hinder the multiple uses of water as it can cause socioeconomic, environmental, and public health impacts, in addition to altering the organoleptic properties of the treated water supply [8,9,10,11]. It is noteworthy that the consumption of aquatic organisms may also be a risk factor because they may bioaccumulate cyanotoxins in their tissues [12,13,14].

In this Northeast Region, there are reports of a death by intoxication in the city of Caruaru, Pernambuco state, which was attributed to water containing cyanotoxins [15]. Another episode of death that stood out occurred in 1996 in a reservoir in the city of Paulo Afonso, Bahia [16].

Cyanobacterial blooms have different toxin-producing and non-toxin-producing species, and in addition, there may be toxic and nontoxic populations of the same species. Therefore, the factors that influence cyanotoxin production, such as genetic regulation and environmental conditions, are still a challenge for researchers [17,18].

In this context, it is important to investigate which organisms are present in the environment and to ascertain whether they have the potential to produce toxins, what toxins they can produce, and whether they are actively producing them [18]. Thus, there is a need to implement reliable analytical methods for the detection and quantification of cyanobacteria and/or their toxins.

Currently, several analytical methods can be used to evaluate the microbiological characteristics of water samples. The main monitoring technique used for the identification and counting of cyanobacteria in reservoirs is optical microscopy; however, for such identification to be viable, an experienced observer with a high level of taxonomic specialization is required. Furthermore, the technique does not allow the inferring of conclusions about the toxicity of the cyanobacteria or the distinguishing of toxic from nontoxic populations [19,20].

For such a distinction to be possible, molecular biology techniques such as polymerase chain reaction (PCR) and sequencing enable the identification of species and their toxigenic potential. An important advance in the field of microbiology was the use of the gene sequence encoding of the 16S rRNA, a molecular “fingerprint” that can be used to identify microorganisms in natural environments. This improvement allowed the development of techniques for the analysis of cyanobacterial biodiversity in natural habitats without the need to carry out a culture step [21]. In turn, the high-performance liquid chromatography (HPLC) technique can be used for the qualitative and quantitative analysis of cyanotoxins and to establish whether these toxin-producing populations are active producers [22,23,24,25].

Thus, to assist in the prevention of toxin exposure from the consumption of water from reservoirs, the study of cyanobacteria and cyanotoxins must resort to various complementary methods in a multidisciplinary manner, to promote a better understanding of the occurrences of the blooms of potentially toxic cyanobacteria [26,27].

Episodes of cyanobacterial blooms have been identified as a problem in many surface water springs, such as the Joanes I Reservoir, which is responsible for 40% of the drinking water supply of the Metropolitan Region of Salvador, Bahia, Brazil. This water body receives the input of effluents along the river course and its waters have been found to have a high nutrient content [28], which can cause phytoplankton proliferation. However, the major concern is the presence of cyanobacteria, which makes it essential to perform a water diagnosis using the various methods and analytical techniques mentioned above [27,29].

The present study investigated the presence of potentially toxic cyanobacterial species, intracellular cyanotoxins, and their specific genes at four sampling points in the Joanes I Reservoir. The monitoring campaign was carried out during the occurrence of phytoplanktonic blooms, with the aim of contributing to the assessment of the conditions of this water body and its proper management.

## 2. Results

### 2.1. Cell Counts

Ten cyanobacteria species/genera were identified at the monitoring sites. The highest total cyanobacterial density at Joanes I Reservoir was observed at P3, which had a concentration of 403,387 cells mL^−1^, followed by P4, with 260,759 cells mL^−1^. At both points, the species responsible for the highest cell density were *Merismopedia tenuissima* and *Romeria heterocellularis* (Table 1). In addition, species/genera of potentially cyanotoxin-producing cyanobacteria were identified at Joanes I Reservoir, such as *Cylindrospermopsis raciborskii* and *Cylindrospermopsis* cf. *acuminato-crispa* and some that were capable of producing microcystin, such as *Aphanocapsa* sp., *Phormidium* sp., and *Pseudanabaena* sp. (Table 1)

### 2.2. Physicochemical and Biochemical Analysis of Water

Table 2 shows the results of the physicochemical analysis of the water samples. It was observed that some parameters, such as total phosphorus, biochemical oxygen demand (BOD), dissolved oxygen (DO), true colour, and chlorophyll a, presented, for all the sampling points, values outside the limits established by the CONAMA Resolution 357/05 [33], which provides the classification of surface water bodies in Brazil. The objective of this classification is to determine the predominant water uses and the adequacy of the pollution controls and to create instruments to evaluate the evolution of the quality of the water bodies. According to this Resolution, class 2 refers to water intended for domestic supply after conventional treatment; for maintaining the protection of aquatic communities; for primary contact recreation; for the irrigation of vegetables and fruit; and for the natural and/or intensive breeding of species intended for human consumption. At point 04, the results of the thermotolerant coliforms showed a value far above (1.2 × 10^4^ UFC/100 mL) the limit standard established by legislation.

**Figure 1:**
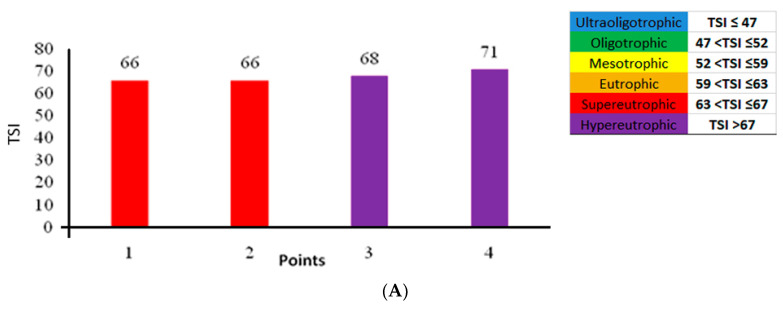

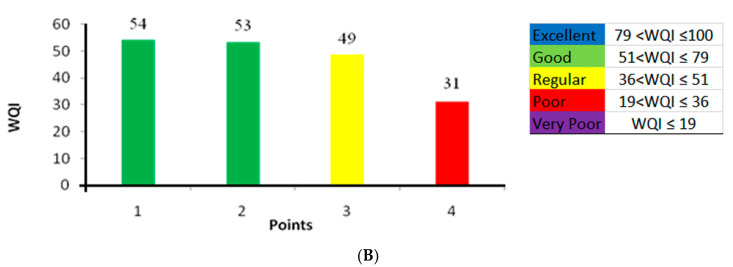
(**A**) Trophic state index (TSI) values for the water samples in Joanes I Reservoir, (**B**) water quality index (WQI) values for the water samples in Joanes I Reservoir.

The sampling points P1 and P2, both with a calculated trophic state index (TSI) of 66, were classified as supereutrophic, while P3 (68) and P4 (71) were classified as hypereutrophic (Figure 1A). The water quality index (WQI) was calculated using nine physicochemical and biological water parameters. P1 and P2 had WQIs of 54 and 53, respectively, and were classified as “good” quality water. P3 had a WQI of 49 and was classified as “regular” quality water, and P4 had a WQI of 31 and was classified as “poor” quality water (Figure 1B).

### 2.3. PCR Analyses

The genetic potential for the production of the microcystin, saxitoxin, and cylindrospermopsin toxins of the cyanobacterial community in the lyophilized samples from the four sampling points was determined by PCR amplification of the *mcy*E (microcystin), *sxt*A (saxitoxin), and *cyr*B (cylindrospermopsin) genes.

Table 3 presents the PCR results. The analyses showed the presence of the microcystin biosynthesis gene *mcy*E with an amplicon of 472 bp for the four sampling sites. The sequencing of the PCR product obtained confirmed the detection of the *mcy*E gene in P3 and P4 (Figure S1).

### 2.4. Toxin Analysis Using LC-MS/MS

The results of the analyses of the cyanotoxins (cylindrospermopsin, anatoxin-a, and homoanatoxin-a) by LC-MS/MS in the lyophilized samples are summarized in Table 4.

The results show the presence of cylindrospermopsin in the P1 and P2 samples and its absence in the P3 and P4 samples (the chromatograms are provided in Supplementary Materials, Figure S2), by the comparison of the retention time and molecular mass with the corresponding standard.

Triplicate analyses of samples P1 and P2 were performed to confirm these results and showed that the 416.3 *m*/*z* peak present at 4 min is well fragmented into a daughter ion of 194.2 *m*/*z*, which indicates that it must be cylindrospermopsin.

The analyses of anatoxin-a and homoanatoxin-a showed their absence in the four sampling points (Figure S3).

## 3. Discussion

### 3.1. Cell Counts

With respect to the taxonomic identification of the cyanobacteria, the species *Merismopedia tenuissima* and *Romeria heterocellularis* had the highest densities at Joanes I Reservoir in the campaign performed. In a campaign performed at P3 at the Joanes I Reservoir by INEMA [34] in February 2015, the species *Merismopedia tenuissima* also had a high density in the sampling (97,339 cells mL^−1^). According to Carmichael [35], in addition to contributing to primary production through photosynthesis, *Merismopedia tenuissima* in high concentrations can also produce lipopolysaccharides, which are known to cause skin irritation on contact and gastrointestinal discomfort.

The species *Romeria heterocellularis* was also found in high densities at the P3 and P4 sampling points. This species was not observed in the campaigns conducted in 2015 at the Joanes I Reservoir by INEMA [34].

These values were much higher than the 50,000 cells mL^−1^ cyanobacterial densities established by Resolution Nr. 357/05 for freshwater—class 2 from the Brazilian National Council on the Environment [33]. In addition, the cyanobacterial cell densities of the P3 and P4 samples exceeded 100,000 cells mL^−1^. This finding indicates that the region around these points in the Joanes I Reservoir reached Alert Level 3 (the highest alert level is for >100,000 cells mL^−1^), which is characterized by the presence of well-defined blooms in the source, with imminent risk to the health of the population [36].

It should be noted that the data on the cyanobacterial densities observed in this study correspond to the rainy season (June 2017) and that higher densities were observed in the campaigns conducted by EMBASA (2015) apud Magalhães [37] in the dry season at the same reservoir’s catchment point (P1), with values of 199,000 cells mL^−1^ (February 2015) and 99,000 cells mL^−1^ (March 2015).

The campaigns performed by INEMA [34], in January and February of 2015 (dry season), also observed high cyanobacteria densities at P1, P2, and P3, the same sampling points adopted in this study. Values ranging from 212,882 cells mL^−1^ to 295,369 cells mL^−1^ were reported, which are higher than those observed in the present study, which involves a rainy season campaign. The exception was P3, where a higher cell density (403,387 cells mL^−1^) was observed in the present study in the rainy season.

Seasonality may affect the physical and biological water parameters, especially the dynamics and concentration of the cyanobacteria [38]. The cyanobacterial density values and rainfall indices analysed at the catchment point of the Joanes I Reservoir, in a monitoring series conducted by INEMA [34] from 2012 to 2015, indicated that the highest cyanobacterial densities were concentrated in the dry months (November to March) in this region. Thus, a low rainfall index may contribute to the concentration of the nutrients derived from external inputs along the basin, such as domestic effluents and fertilizer runoff, among others, contributing to cyanobacterial blooms and, consequently, water quality degradation [39].

In the present study, low densities of *Cylindrospermopsis raciborskii* were observed at the P1, P2, and P3 sampling points. However, according to the results of the monitoring plan conducted by INEMA [34] from February to June 2015 at the sampling point (P1) of the Joanes I Reservoir, the presence of the *Cylindrospermopsis raciborskii* species was observed in all the campaigns, with highest density in March and April and a sharp reduction in May and June. In the qualitative and quantitative analyses of cyanobacteria performed by INEMA [34] at P1, P2, and P3, *Cylindrospermopsis* sp. was one of the most predominant species in all the sampling points and campaigns of the study.

Several studies show the recurrent records of the presence and dominance of *Cylindrospermopsis raciborskii* in the waters of the reservoirs and dams in the several states of the Northeast Region of Brazil. In addition to this species, the occurrence of *C. philippinensis*, *C. catemaco*, and *C. acuminato*-*crispa* was also recorded [15,40].

The genus *Cylindrospermopsis* has been widely studied, and some of its species are of sanitary importance due to the formation of blooms, their wide distribution around the world, and the capacity to produce cyanotoxins (saxitoxins and cylindrospermopsins). In Brazil, *C. raciborskii* is widely distributed and found in water bodies throughout the country [30]. The increased occurrence of this species in reservoirs in Brazil, mainly in the Northeast Region, is due to its adaptive advantages. The presence of gas vacuoles allows the individuals to migrate in the water column, going to the bottom of the water body to use the available organic nitrogen and to remain on the surface where they can capture radiation, which is more intense in that region, and even fix atmospheric nitrogen [31].

The species *Aphanocapsa delicatissima* was present at the four sampling points, with a cell density ranging from 8806 to 20,230 cells mL^−1^. In the campaign performed by INEMA [34] in the Joanes I Reservoir, conducted in January 2015, the species *A. Delicatissima* was found at a density of 8124 mL^−1^ cells at P1 and 30,466 cells mL^−1^ at P3. According to Komárek and Anagnostidis (1998), this species is cosmopolitan. In Brazil, species of the genus *Aphanocapsa* were found as producers of microcystin [32].

In the present work, the genus *Phormidium* sp. was detected at the P1 and P2 sampling points in very low densities. Some of its species have the ability to produce microcystins, saxitoxins, anatoxin-a (ATX), and homoanatoxin-a (HTX) [31]. In the campaigns performed by INEMA in 2015 [34] in January and February, these species were not recorded. The species of *Pseudonabaena* sp. are potential producers of microcystins and stand out in blooms [26] and were also detected in the present work.

### 3.2. Physicochemical and Biochemical Analysis of Water

The values of the total phosphorus, biochemical oxygen demand (BOD), dissolved oxygen (DO), true colour, and chlorophyll *a* parameters at all the sampling points were outside the limits established by CONAMA Resolution Nr. 357/05 for freshwater—class 2 [3352]. At P4, the thermotolerant coliforms had a much higher value (1.2 × 10^4^ CFU/100 mL) than the limit established by the legislation. This parameter is considered a bioindicator of water contamination by domestic sewage since the presence of thermotolerant coliforms is directly related to the degree of faecal contamination.

The total phosphorus values were above the limits established (<0.03 mg L^−1^ for lentic environments) by the aforementioned legislation at the four sampled points, as shown in Table 2. The highest total phosphorus values were observed at the P3 (0.15 mg L^−1^) and P4 sampling points (0.25 mg L^−1^), which may have contributed, in addition to other factors, to the elevated cyanobacterial density of 403,387 cells mL^−1^ and 260,759 cells mL^−1^ at the respective points. The chlorophyll concentrations at the sampling points ranged from 31 μg L^−1^ to 54 μg L^−1^ and were above the limit (≤30 μg L^−1^) established by CONAMA Resolution Nr. 357/05 [33]. The highest concentration was observed at P4 (54 μg L^−1^), which was accompanied by a high cyanobacterial density of 260,759 cells mL^−1^. This value is much higher than the one established by the resolution (<50,000 cells mL^−1^), which may be a direct result of the eutrophication of the Joanes I Reservoir, as indicated by the calculated TSI, classified as hypertrophic at P4 (Figure 1A).

In the present study, the waters of the Joanes I Reservoir presented eutrophic conditions, as expected, mainly because it receives the inflow of domestic effluents without treatment along the course of Joanes river. The physicochemical and biological data indicate degradation of the water quality of this water body.

It is worth noting that the sampling was performed in July, when the rainfall index is the highest, even though the points sampled in the reservoir showed some parameters outside the standards established by the current legislation [33,41,42]. The total phosphorus and chlorophyll *a* parameters are indicators of the trophic state and have a direct relationship with the high cyanobacterial densities (>50,000 cells L^−1^) encountered at the P3 and P4 sampling points.

The total phosphorus content observed in previous studies [28,33,37] was also above that permitted by the legislation, ranging from 0.05 mg L^−1^ to 0.25 mg L^−1^ for samples collected at different times in 2015 at the Joanes I Reservoir. With respect to the chlorophyll *a*, high values were observed in the campaign performed by INEMA [34], coinciding with high cyanobacteria densities, especially at the P4 sampling point, which is the receiving point of effluents released in the area of the Joanes I Reservoir. Ordinance no. 888/2021 of the Brazilian Ministry of Health [42] recommends monitoring the chlorophyll *a* parameter weekly as an indicator of the potential for increasing cyanobacterial density.

Dissolved oxygen (DO) is the most important indicator of environmental quality because it is essential for the maintenance of the vital processes of aquatic organisms. The DO values at all the sampling points were outside the limits established by CONAMA Resolution no. 357/05, as shown in Table 2, with values lower than 4.0 mg L^−1^. The lowest value was observed at the P4 sampling point (2.48 mg L^−1^). In natural waters, oxygen is indispensable for living organisms, especially fish, and most species cannot withstand a DO concentration in the water below 4.0 mg L^−1^ [43]. DO values lower than 4.6 mg L^−1^ were also found during campaigns conducted in February, March, and September 2015 [33], in a campaign conducted in January 2015 [28], and in a campaign conducted from March to June 2015 [37]. Decreased oxygen concentration in aquatic environments may be a result of high temperatures, which shift the balance, generating increasing losses of dissolved oxygen to the atmosphere, organic matter mineralization, ion oxidation, and the presence of biodegradable organic pollutants, where the bacteria originally present in the water degrade these compounds and consume the DO in the water [44].

The BOD_5_ values were above the limits established by CONAMA Resolution Nr. 357/05 for fresh water—class 2 (<5 mgO_2_ L^−1^) [33] at all the sampling points of the present study. These BOD_5_ results may be due to a high concentration of organic matter in the waters of the Joanes I Reservoir at the sampled points, caused by the presence of organic load and decomposer microorganisms, which can reduce DO concentrations in water.

A general analysis of the physicochemical and biological parameters of the waters of the Joanes I Reservoir shows that P3 and P4 had higher values than the limits established by the legislation when compared with the other sampling points. The high TSI values are related to the high total phosphorus and chlorophyll *a* values, which, together with the DO, BOD, and thermotolerant coliforms values outside the limits required by the legislation, indicate that the Joanes I Reservoir receives domestic effluent and is eutrophicated. The effluents dumped in the reservoir, near P3 and P4, come from the cities of Camaçari and Simões Filho and from the communities in these municipalities. This compromises the multiple use of the reservoir, mainly due to the excess nutrient load to its waters and, consequently, the proliferation of potentially toxin-producing cyanobacteria.

The results obtained for the WQI were calculated using nine physicochemical and biological water parameters. Points P1 and P2 had WQIs of 54 and 53, respectively, and were thus classified as “good” quality water, and these values were close to the lower limit of the range established for “good” water quality of 51 to 79. These results were influenced by the low DO values and by the presence of thermotolerant coliforms in P1 (30 CFU/100 mL) and P2 (19 CFU/100 mL). According to the legislation [42], faecal microorganisms should not be present in waters intended for human consumption. Although the presence of coliforms was identified, the relatively low concentration possibly occurred due to the release of clandestine effluents downstream of the catchment point. Ferreira and Almeida [45] indicate that the WQI decreases even with small concentrations of these microorganisms.

In the campaigns conducted by INEMA [34] in March and September 2015 and by EMBASA (2015) apud [37], where the average values were calculated over a historical series from 2006 to 2014 for the WQI parameter at the sampling point P1, the water of the Joanes I Reservoir was classified as having “good” quality.

In the present study, point P3 had a WQI of 49, which classifies the water quality as “regular”, and point P4 had a WQI of 31, which classifies it as “poor” quality water (Figure 1B). Possibly, the WQI of P4 is due to the high concentration of thermotolerant coliforms, a variable that exceeded the limit established in Ordinance no. 888/21 [42], which shows, once again, the influence of the disposal of domestic effluents without previous treatment in the region. Furthermore, the low DO concentration also contributed to a “poor” water quality level.

### 3.3. PCR Analyses

The detection of the *mcy*E gene in all the samples from Joanes I Reservoir indicates that the cyanobacterial species present in this water body have the ability to synthesize microcystin. However, to confirm its expression, it is necessary to carry out biochemical analyses (LC/MS and Elisa, among others) to verify whether the toxic strains are in active production [18,46]. In the present work, microcystin analysis was not performed by LC/MS because the methodology was not implemented in the available equipment. However, the set of data obtained was sufficient to meet the proposed objectives.

Although some genera related to the production of microcystins were identified at the sampling points, the positive results for the presence of the specific gene encoding microcystin (Table 3), specially at the points P3 and P4, may be related to the genus *Aphanocapsa*, which was observed in greater quantity at these points (Table 1) [26,32,40].

The genes associated with the production of cylindrospermopsin (*cyr*B) and saxitoxin (*stx*A) were not detected in any of the samples by PCR analysis. Regarding cylindrospermopsin, its presence was evidenced, mainly at points P1 and P2, by the formation of bands with an amplicon at around 597 bp (Figure S4). Perhaps the sequencing did not give a positive result due to the small quantity of genetic material present in the bands.

In addition to the species identified as potential producers of cyanotoxins being recorded by optical microscopy, which is the main technique used in Brazil to monitor these organisms, it is recommended to implement complementary techniques to improve the study and monitoring of these microorganisms in reservoir waters intended for public supply. There are no morphological differences between the toxic and nontoxic populations. Thus, the preventive and systematic assessment of toxic cyanobacteria in water resources is advisable to avoid contamination and public health problems. The prediction of toxic blooms is very important, given their increasing occurrence in large water supply systems and the high cost of the current technology used for their removal.

### 3.4. Toxin Analysis Using LC-MS/MS

The presence of cylindrospermopsin was identified by LC/MS at the sampling points P1 and P2 only. The presence of this toxin was not confirmed at P3 and P4, which suggests that it was not being produced or was present at concentrations below the detection limit of the analytical method. Previous studies refer to variations occurring as a function of environmental parameters such as light, temperature, pH, and nutrients, which may influence the production of cyanotoxins [31].

According to the Brazilian Ministry of Health Ordinance Nr. 05/2017, only the monitoring for microcystin and saxitoxin is required. Cylindrospermopsin and anatoxin-a are analysed only when the presence of the cyanobacterial genera potentially producing these toxins is detected [41]. However, the ordinance was updated in 2021 and analyses for cylindrospermopsin became mandatory [42].

Despite not having performed a quantitative analysis of the cyanotoxins, the present study presented quantitative data on several environmental parameters, including the density of the cyanobacteria, which in two sample points exceeded the acceptable values established by the regulations. Moreover, ten cyanobacteria species/genera were identified at the monitoring sites, including five potentially cyanotoxin-producing species, and a positive result for the presence of the cylindrospermopsin toxin was confirmed by LC-MS/MS, which indicated that the cyanobacteria populations are actively producing toxins, and analysis of the PCR products using the HEPF/HEPR primer pair for the detection of the microcystin was positive for the analysed samples. This information by itself can contribute to the planning of preventive actions and the minimising of the impacts on public health.

## 4. Conclusions

Potentially toxic cyanobacteria were identified in the Joanes I Reservoir water samples, which were classified as having a high potential for the excessive growth of these microorganisms. The high TSI values and the high concentrations of total phosphorus and nitrogen, together with low DO and high BOD values, indicate that this reservoir received input from domestic and/or industrial effluents and has a high eutrophication potential.

These data are worrisome because the molecular detection of microcystin genes at the four sampling points indicated the possible potential for the production of these toxins in the whole water body. Toxin analyses by HPLC confirmed the presence of cylindrospermopsin in the P1 and P2 sampling points, which suggests that they were possibly produced by *Cylindrospermopsis raciborskii*. The presence of this toxin was not confirmed at P3 and P4, which suggests that it was not being produced or was present at concentrations below the detection limit of the analytical method. Since 2021, the analyses of cylindrospermopsin for monitoring the water quality for public supply became mandatory in the new Brazilian legislation, due to the high risk to human health.

The molecular technique using PCR can detect toxic genotypes at the beginning of the growth stage, long before the toxic cyanobacterial blooms are formed, thus becoming a predictive technique; it is important for this to be implemented in monitoring programs by water management agencies.

In addition to the species identified as potential producers of cyanotoxins by the optical microscopy method, it is important to implement complementary analytical methods such as molecular biology techniques to improve the study and monitoring of these microorganisms in reservoir waters intended for public supply and thereby avoid public health problems.

This is the first study conducted at the Joanes I Reservoir in which a molecular biology technique (PCR) was used in conjunction with other analytical techniques for the study of cyanobacteria and cyanotoxins. Studies such as this one can support actions to prevent the exposure of living beings to cyanotoxins.

It is important to jointly use analytical methods and techniques in a multidisciplinary manner, where each complements the others and contributes to the planning of preventive actions and the minimising of impacts, especially in water bodies that are intended for multiple uses and human supply, as is the case with the Joanes I Reservoir.

## 5. Materials and Methods

### 5.1. Sampling Sites

The Joanes I Reservoir is an important water supply source for several municipalities in the state of Bahia, Brazil, including Salvador, Candeias, Lauro de Freitas, Simões Filho, Dias D’Ávila, Madre Deus, and São Francisco do Conde, and it is also used for aquaculture, animal drinking, recreation, fishing, and navigation.

**Figure 2:**
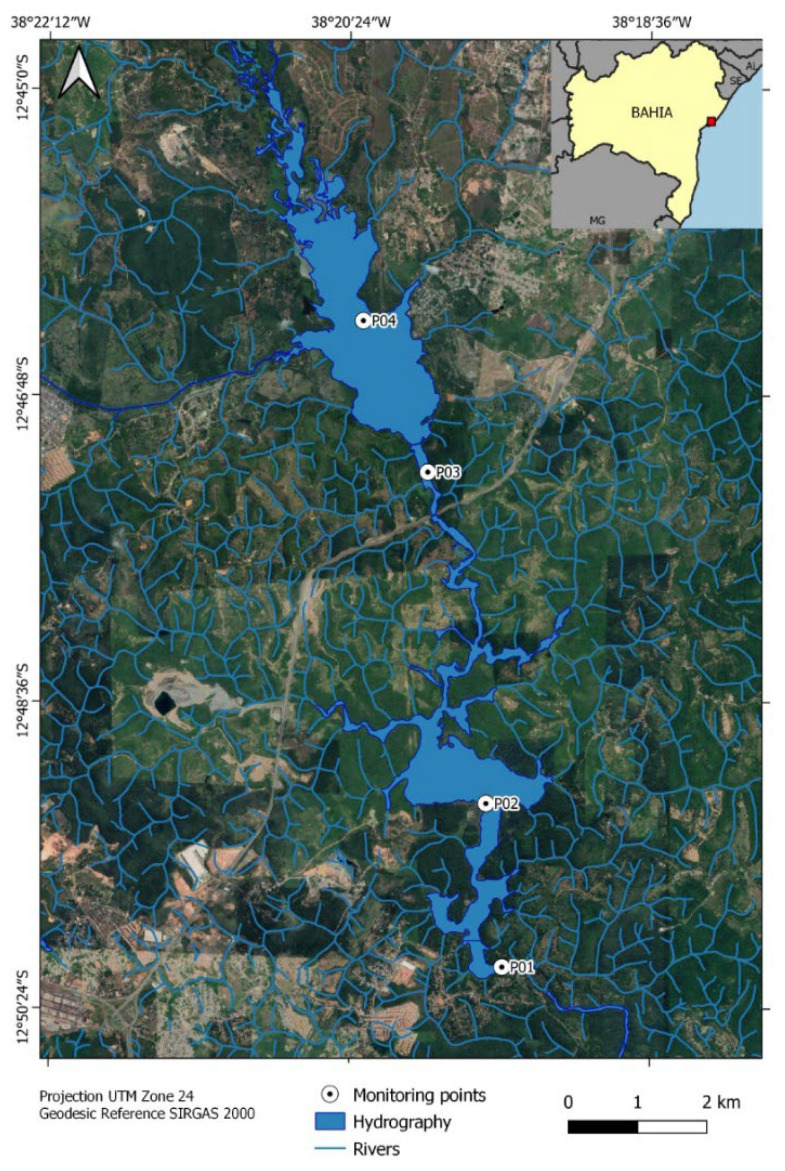
Map of the location of the sampling points at Joanes I Reservoir.

For the present study, water analysis was performed in a monitoring campaign during the period of phytoplankton bloom occurrence at Joanes I Reservoir on 6 July 2017. The water samples were collected at four different points in the Joanes I Reservoir (P1, P2, P3, and P4). The location, coordinates, and description of the sampling points are shown in Figure 2 and Table 5. The sampling points used in this study followed the same monitoring points used by the concessionaire Bahia Water and Sanitation Company (Empresa Baiana de Águas e Saneamento—EMBASA) of Bahia, Brazil.

### 5.2. Cell Counts

The samples collected in a 30-micron net were preserved in Lugol’s solution and sent for a cyanobacterial cell count and species identification by the optical microscopy at the Laboratory of Ecology and Paleoecology—EcoPaleo. The Utermöhl method was used, the analysis methodology of which was based on the Methods for Quantitative Phytoplankton Analysis manual [47]. The percentage of error of these measurements is estimated to be 10% as 400 individuals were counted in each analysis.

### 5.3. Physicochemical and Biochemical Analysis of Water

Water samples were collected approximately 30 cm below the water depth in plastic containers. The temperature, dissolved oxygen (DO), conductivity, and pH parameters were measured in the field using a calibrated multiparameter probe (HANNA HI 9828, Woonsocket, RI, USA), with an estimated measurement error of 15%. The laboratory analyses were performed by standard methods [48] with an estimated error of 10%.

Calculation of the Water Trophic State Index and Water Quality Index

The water trophic state index (TSI) is a measure of the eutrophication potential, and it was calculated from the phosphorus and chlorophyll a value [49] according to the equations:

TSI (CL) = 10 × (6 − (((0.92 − 0.34 × (ln(CL)))/ln2))(1)

TSI (PT) = 10 × (6 − ((1.77 − 0.42 × (ln(PT))/ln2))(2)

TSI = [TSI (CL) + TSI (PT)]/2(3)

where

PT: total phosphorus concentration measured at the water surface, in μg L^−1^;

CL: chlorophyll a concentration measured at the water surface, in μg L^−1^;

ln: natural logarithm.

The TSI values are classified according to trophic state classes [49,50] and shown in Table 6.

The water quality index (WQI) was also calculated and set as a function of its importance to the overall conformation of the water quality. The WQI, adapted from the National Sanitation Foundation of the USA, was determined using nine variables: water temperature, pH, dissolved oxygen, biochemical oxygen demand, thermotolerant coliforms, total nitrogen, total phosphorus, total solids, and turbidity. The methodologies concerning each individual variable with their respective weights are detailed in [49] and presented in the Supplementary Materials. The WQI calculation and classification values followed a range from 0 to 100 (the higher the value the better the water quality) and are shown in Table 7.

The WQI values are classified into ranges that vary between Brazilian states [49,51].

### 5.4. PCR Assay for Cyanotoxin Genes

The samples collected in amber bottle were filtered through 0.47-micron fiberglass filters and stored in a freezer (−20 °C). They were then lyophilized for 14 h in an Enterprise 1 lyophilizer (Terroni) and analysed by molecular biology. The analytical procedures included DNA extraction, PCR amplification, electrophoresis, and sequencing. These steps are described below.

#### 5.4.1. Genomic DNA Extraction

Lyophilized material was used for DNA isolation. The first extraction step began with cell lysis of the cyanobacteria in a microtube, adding 300 μL of TE buffer (Tris EDTA—pH 7.8 for DNA), glass beads (approximately 100 μL), and 300 μL of the phenol:chloroform:isoamyl alcohol mixture (25:24:1) (Roth). Then, the microtubes were placed in a cell disruptor (FastPrep-24™ 5G, MP Biomedicals, Eschwege, Germany). After this procedure, the microtubes were centrifuged for 5 min at 4200× *g*, and the supernatant was removed and transferred to new 1.5 mL microtubes. For DNA precipitation, 100% ethanol was added until completing a volume of 1.5 mL. Then, the microtubes were inverted 10× and centrifuged for 20 min at 4200× *g*, and the supernatant was discarded. The same procedure was used with 70% ethanol, with centrifugation for 2 min. After the ethanol was discarded, the microtubes were placed in a heating block at 50 °C for residual ethanol evaporation, and then, 50 μL of Milli-Q water was added.

#### 5.4.2. PCR Amplification of Genes

For each pair of cyanotoxin-specific primers (Table 8), PCRs were performed in a final volume of 20 μL. The PCR mixture contained 10 μL of 2× Red Taq Master Mix (mixture containing Taq DNA Polymerase, dNTPs, MgCl_2_, and reaction buffer at ideal concentrations for efficient DNA amplification by PCR), 2 μL of template (DNA extract or ultrapure water), 1 μL of each primer (cylindrospermopsin, saxitoxin, and microcystin), and 6 μL of ultrapure water. The template used for the negative control (−) was ultrapure water.

#### 5.4.3. Electrophoresis

The presence of DNA fragments after PCR was verified by electrophoresis in a 1% agarose gel diluted in TAE buffer. For 30 mL of agarose, 1.8 μL of the stain Midori Green (Advance DNA Stain, Nippon Genetics Europe, Düren, Germany) was added to a horizontal gel apparatus (PeQlab, Erlangen, Germany). Five microliters of the marker (1 kb DNA Ladder, New England BioLabs, Ipswich, MA, USA) and 10 μL of PCR products were added to each well. The running buffer used was 1× TAE, and the tension used was 120 volts. The DNA bands in the gel were visualized under UV light in a transilluminator (Phase), and photographs were taken with a Cannon camera.

#### 5.4.4. Band Extraction and Sequencing

The bands resulting from the PCR amplification were excised from the gel, and the DNA was extracted with the DNA Gel Extraction kit (Monarch, New England BioLabs, Ipswich, MA, USA), following the manufacturer’s instructions. After extraction, the purified DNA was sent for sequencing at GATC Biotech. Sequence identity was determined by a BLAST search of the National Center for Biotechnology Information (NCBI) database.

### 5.5. Analysis of Intracellular Cyanotoxins/Toxin Using LC-MS/MS

The water samples for the analysis of cyanotoxins (cylindrospermopsin, anatoxin-a, and homoanatoxin-a) were filtered through 0.47-micron fiberglass filters and then lyophilized. The extraction of toxins from the biomass on the lyophilized filter comprised the following steps: 10 mL of 0.1% formic acid was added to the filters, which were left for 24 h at room temperature and occasionally vortexed. The material was transferred to a Corex glass tube and sonicated with an ultrasonic probe (Misonix Ultrasonic Liquid Processors, Farmingdale, NY, USA) at 40% amplitude for 2 min. The material was then centrifuged at 4200× *g* for 30 min at 25 °C. Next, the supernatant was removed with a syringe and needle and passed through a 0.2 μm polyether sulfone filter (Nalgene, Darmstadt, Germany) to a 1.5 mL vial, and 5 μL of the sample was injected into the HPLC system for analysis.

An Atlantis dC18 reverse-phase column (150 × 2.1 mm, 3 μm) was used. The eluent used for the analysis of cyanotoxins in the extracts was methanol (Hipersolv Chromanorm, VWR Chemicals BDH) and Milli-Q water (0.1% formic acid) at a flow rate of 0.200 mL/min and in gradient mode according to Mann et al. [57].

For LC-MS/MS analysis, two systems were used, the first consisting of a tandem mass spectrometer (6410 series, Agilent Technologies, Santa Clara, CA, USA) coupled to an electrospray ionization (ESI) source in the Agilent LC system (1200 Series, Agilent, Santa Clara, CA, USA) and equipped with an automatic sampler (MicroWPS 1200 Series, Agilent, Santa Clara, CA, USA). The analytes were detected in a triple quadrupole instrument in a multiple reaction monitoring (MRM) mode. The second system used was a Varian 320-MS quadrupole tandem mass spectrometer coupled to an ESI source of the Varian LC system (212-LC, Varian, Palo Alto, CA, USA) and equipped with an auto sampler (Prostar 410, Varian, Palo Alto, CA, USA). The data were acquired in positive ion mode [57,58].

## Figures and Tables

**Table 1:** Cyanobacterial density (cells mL^−^^1^) at the sampling points in Joanes I Reservoir.

Cyanobacterial Species	P1	P2	P3	P4	Cyanotoxins	Reference
*Sphaerocavum brasiliense*	60	24	476	0	-	
*Cylindrospermopsis* cf. *acuminato-crispa*	24	6	952	60	saxitoxins, cylindrospermopsins	[30]
*Cylindrospermopsis raciborskii*	78	354	12	0	saxitoxins, cylindrospermopsins	[30]
*Phormidium* sp.1	12	6	0	0	microcystins, saxitoxins, anatoxin-a, homoanatoxin-a	[31]
*Aphanocapsa delicatissima*	13,209	8806	16,184	20,230	-	
*Aphanocapsa* sp.1	476	238	5236	5950	microcystin	[32]
*Merismopedia glauca*	0	0	0	7735	-	
*Merismopedia tenuissima*	357	6426	283,900	112,455	-	
*Pseudanabaena* sp.1	238	238	0	90	microcystin, anatoxin-a	[26]
*Romeria heterocellularis*	17,255	952	96,628	114,240	-	
**Total**	31,709	17,050	403,388	260,759		

**Table 2:** Physicochemical and biochemical parameters of the water in Joanes I Reservoir.

Parameter	CONAMA Resolution 357/05 Freshwater—Class 2	Unit	P1	P2	P3	P4
NUTRIENTS						
Total nitrogen N		mg L^−1^	3.83	1.05	3.27	3.98
Total phosphorus P	≤0.03	mg L^−1^	0.05 *	0.06 *	0.15 *	0.25 *
Nitrate N-NO_3_	≤10	mg L^−1^	0.10	0.11	0.85	0.58
Nitrite N-NO_2_	≤1.0	mg L^−1^	0.01	0.01	0.13	0.23
Ammonia N-NH_4_	≤3.7 mg L^−1^ N for pH <7.5	mg L^−1^	0.35	0.33	0.45	0.98
Phosphate P-PO_4_		mg L^−1^	0.00	0.00	0.00	0.12
SYSTEM CONSTITUENTS						
Total solids		mg L^−1^	92.0	91.0	107	129
COD		mg L^−1^ O_2_	26.80	24.50	29.82	32.47
BOD	≤5 mg L^−1^ O_2_	mg L^−1^ O_2_	7.15 *	5.23 *	13.93 *	19.71 *
True colour	≤75 mg Pt/L	mg Pt/L	99 *	81 *	170 *	183 *
Turbidity	≤100 NTU	NTU	2.82	2.58	5.53	7.84
Temperature		°C	26.6	27.3	28.4	27.9
pH	6.0 to 9.0		7.04	6.76	6.5	6.47
Dissolved oxygen DO	≥5	mg L^−1^ O_2_	3.77 *	2.53 *	3.22 *	2.48 *
Electric conductivity		µs cm^−1^	218	319	223	238
Redox potential		ROP	−42.5	−54.1	−38.2	−36.7
Total dissolved solids	≤500 mg L^−1^	mg L^−1^	109	109	111	119
Secchi Disk Transparency		M	1.03	1.12	0.98	0.72
MICROBIOLOGICAL CONSTITUENTS						
Chlorophyll *a*	≤30 µg L^−1^	mg/m^3^	45 *	42 *	31 *	54 *
Thermotolerant coliforms	≤1000	UFC/100 mL	30	19	120	12,000 *
Cyanobacterial density	≤50,000	cells mL^−1^	31,709	17,050	403,387 *	260,759 *

* Values above of the standard established in CONAMA Resolution no. 357/05 for freshwater class 2 [33].

**Table 3:** Summary of the results obtained from the PCR analyses for the water samples in Joanes I reservoir, indicating the genes and the primers used.

Samples	Microcystin	Saxitoxin	Cylindrospermopsin
	*mcy*E (HEPF/HEPR)	*sxt*A (saxtaF/saxtaR)	*cyr*B (M13/M14)
P1	(+)	(−)	(−)
P2	(+)	(−)	(−)
P3	(+)	(−)	(−)
P4	(+)	(−)	(−)
Negative control	(−)	(−)	(−)

Legend: (+) positive PCR result; (−) negative PCR result.

**Table 4:** Results obtained by LC/MS/MS performed on the lyophilized samples at the sampling points.

Sampling Points	Anatoxin-A and Homoanatoxin-a	Cylindrospermopsin
P1	(−)	(+)
P2	(−)	(+)
P3	(−)	(−)
P4	(−)	(−)

Legend: (+) Presence; (−) Absence.

**Table 5:** Location of the sampling points.

Geographic Coordinates		
Code	Latitude (S)	Longitude (W)
P1	12°50′9.37″	38°19′29.0″
P2	12°49′11.73″	38°19′34.90″
P3	12°47′15.01″	38°19′56.05″
P4	12°46′21.68″	38°20′19.33″

Source: EMBASA, 2017 [37].

**Table 6:** Trophic state index classification for reservoirs.

TSI Classification	Weighting
Ultraoligotrophic	TSI ≤ 47
Oligotrophic	47 < TSI ≤ 52
Mesotrophic	52 < TSI ≤ 59
Eutrophic	59 < TSI ≤ 63,
Supereutrophic	63 < TSI ≤ 67
Hypereutrophic	TSI > 67

Source: CETESB, 2022 [49].

**Table 7:** Water quality index.

WQI Classification	
Category	Rating
Excellent	79 < WQI ≤ 100
Good	51 < WQI ≤ 79
Regular	36 < WQI ≤ 51
Poor	19 < WQI ≤ 36
Very Poor	WQI ≤ 19

Source: CETESB, 2022 [49]; ANA, 2016 [51].

**Table 8:** List of primers used for qualitative PCR.

Gene	Primer	Primer Sequence (5′–3′)	Bp	Reference
*mcy*E	HEPF	TTTGGGGTTAACTTTTTTGGGCATAGTC	472	[52]
	HEPR	AATTCTTGAGGCTGTAAATCGGGTTT		
*sxt*A	saxtaF saxtaR	GCGTACATCCAAGCTGGACTCG GTAGTCCAGCTAAGGCACTTGC	600	[53,54]
*cyr*B	M13 M14	GGCAAATTGTGATAGCCACGAGC GATGGAACATCGCTCACTGGTG	597	[55,56]

## Data Availability

Not applicable.

## Supplementary Materials

The following supporting information can be downloaded at: https://www.mdpi.com/article/10.3390/toxins15010051/s1, Figure S1: sequences of the PCR products with the primers HEPF/HEPR (microcystin) from points 3 and 4, Figure S2: LC/MS analyses of cylindrospermopsin (CYN) at the four sampling points from Joanes I Reservoir, Figure S3: chromatograms for anatoxin-a and homoanatoxin-a search at the four sampling points from Joanes I Reservoir, Figure S4: PCR analysis using the M13/M14 primers (597 bp); methodology for water quality index (WQI), Table S1: Variables and relative weights used in NSF WQI and CETESB WQI calculus.
