# Supplementary material for: Identification of Cyanobacteria and Its Potential Toxins in the Joanes I Reservoir, Bahia, Brazil
Source: Toxins (Basel). 2023 Jan 6;15(1):51. doi: 10.3390/toxins15010051 (PMC9865514; doi:10.3390/toxins15010051)
Supplement: Supplementary file 1 [file toxins-15-00051-s001.zip › toxins-1945446-supplementary.pdf]

Supplementary Materials:

Manuscript:

"Identification of Cyanobacteria and its Potential Toxins in the Joanes I Reservoir, Bahia, Brazil"

(Point 3):
GenBank: MW602420.1
>MW602420.1 Uncultured *Microcystis* sp. clone Kop 7 microcystin synthetase (*mcy*E) gene, partial cds
TTTGGGGTTAACTTTTTTGGGCATAGTCCGGATTTTGTCATTGAAGCGGTTCAAGAACAAATGAACCGAG
GAATAAGTTTAGGAATGCAGTCAAATCTGGCTGCCGAAACCGCCGTTTTTAATTAGTGAAATGGGTCGAGT
CGAAAGAGTCGCTTTTAGTAATACGGGAACCGAGGCGATATGGCCGCTGTTCGCATTGCTCGCTCTCGT
ACAAAACGTCAAAAAATCGTTATGTTCGCTGGCTCCTACCATGGAACTTTTGACGGCATCTTAGCACGAG
TAGGAGAAGATAAAACCACGACTCAACCCTTAAGTTTAGGCACTCCTTTAGGAATGGTTGAAGACATAAT
AGTCTTGAGTTATGGAGTTGAAGAAAGCCTCGATATTATTGCTACTCATGCTGATGATTTAGCCGCCGTA
TTAGTCGAACCAGTTCAAAGTCGCAAACCCGATTTACAGCCCAAGAATT

(Point 4):
GenBank: FJ429841.2
>FJ429841.2 Uncultured *Microcystis* sp. from Uganda microcystin synthetase (*mcy*E) gene, partial cds
TTTGGGGTTAACTTTTTTGGGCATAGTCCGGATTTTGTCATTGAAGCGGTTCAAGAACAAATGAACCGAG
GAATAGGTTTAGGAATGCAGTCAAATCTGGCTGCCGAAACCGCCGCTTTAATTAGTGAAATGGGTCGAGT
CGAAAGAGTCGCTTTTAGTAATACGGGAACCGAGGCGATTATGGCCGCTGTTCGCATTGCTCGCTCTCGT
ACAAAACGTCAAAAAATCGTTATGTTCGCTGGCTCCTACCATGGAACTTTTGACGGCATCTTAGCACGAG
TAGGAGAAGATAAAACCACGGCTCAACCCTTAAGTTTAGGCACTCCTTTAGGAATGGTTGAAGACATAAT
AGTCTTGAGTTATGGAGTTGAAGAAAGCCTCGATATTATTGCTACTCATGCTGATGATTTAGCCGCCGTA
TTAGTCGAACCAGTTCAAAGTCGCAAACCCGATTTACAGCCTCAAG

Figure S1: Sequences of the PCR products with the primers HEPF/ HEPR (microcystin) from points 3 and 4.

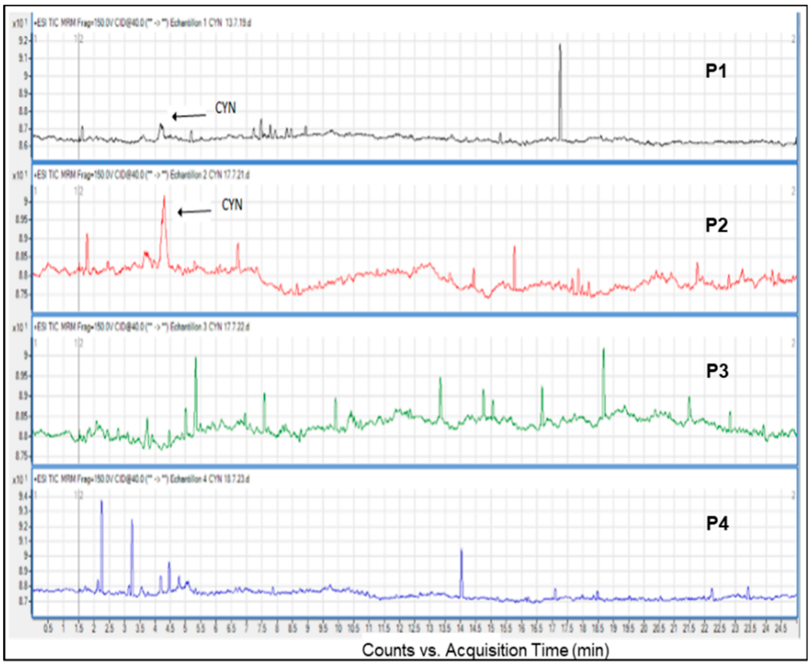

Figure S2: LC/MS analyses of cylindrospermopsin (CYN) at the four sampling points from Joanes I Reservoir.

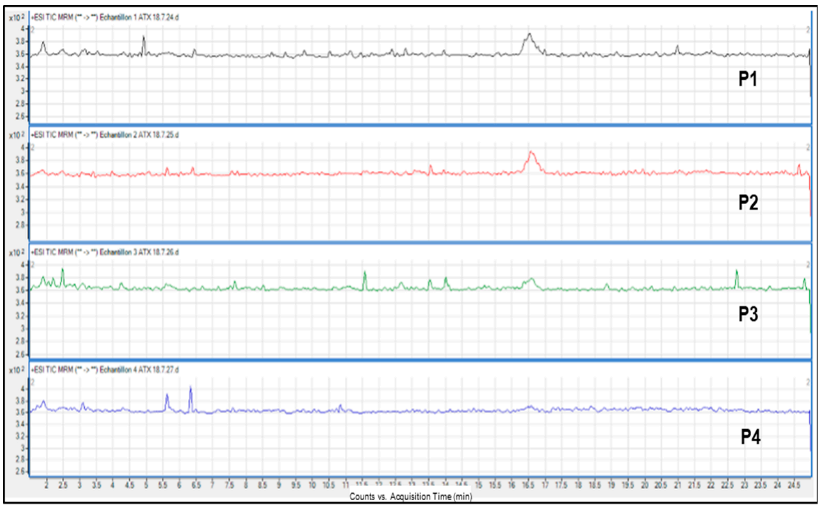

Figure S3: Chromatograms for anatoxin-a and homoanatoxin-a search at the four sampling points from Joanes I Reservoir.

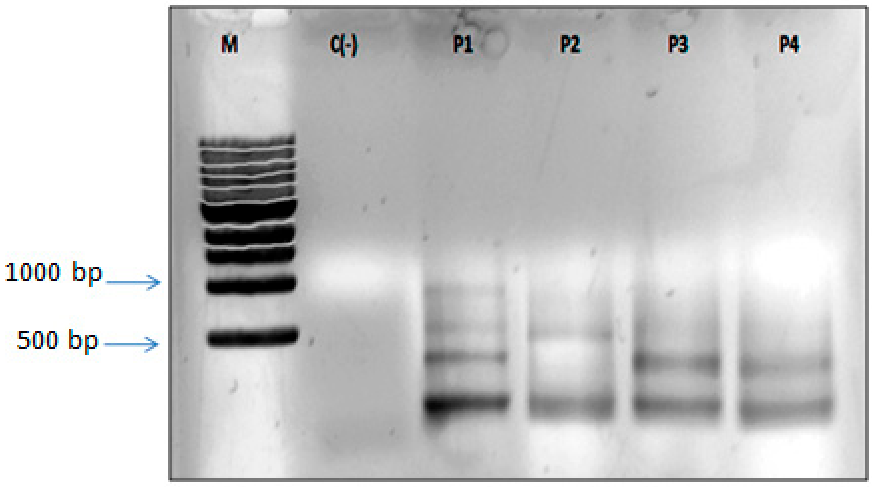

Figure S4. PCR analysis using the M13/M14 primers (597 bp). M: 1 kb marker; C(-): negative control; columns 3 to 6 correspond to P1 to P4.

Methodology for Water Quality Index (WQI):

The Brazilian WQI is an adaptation from the NSF index. The Environmental Company of the State of São Paulo (CETESB)* adapted the National Sanitation Foundation NSF WQI. Of the 35 variables initially considered, the index incorporates the 9 deemed to be most relevant for water quality evaluation, having as main concern its use for public drinking water supply. It is computed as the weighted product (Eq. (1)) of the normalized values of nine variables: temperature, pH, dissolved oxygen (DO), biochemical oxygen demand (BOD), thermotolerant coliforms (TC), dissolved inorganic nitrogen (DIN), total phosphorus (TP), total solids (TS) and turbidity (T).

$$WQI = \sum qi\ Wi / \sum Wi \qquad (1)$$

Where:

- WQI: Water Quality Index
- qi: is a relative value of water quality parameter
- i: is a number of parameters that are taken into account
- Wi: is a factor that calculates parameter significance

.

Each parameter is weighted by a value *Wi* between 0 and 1 and the sum of all weights is 1. The result is expressed by a number between 0 and 100, divided in 5 quality ranges: (100–79)—excellent quality; (79–51)—good quality; (51–36)—regular quality; (36–19)—poor quality; (19–0)—very poor quality. Normalization curves for each variable, as well as the respective weights, are available in CETESB site*.

Table S1 Variables and relative weights used in NSF WQI and CETESB WQI calculus

Variables	Relative weight (WQI -NSF)	Relative weight (WQI - CETESB)
Dissolved oxygen	0.17	0.17
Thermotolerant coliforms	0.16	0.15
Hydrogen ionic potential	0.11	0.12

Biochemical oxygen demand	0.11	0.10
Phosphorus	0.10	0.10
Water temperature	0.10	0.10
Nitrogen	0.10	0.10
Turbidity	0.08	0.08
Total residues	0.07	0.08

*CETESB - Environmental Company of the State of São Paulo - CETESB. *Inland water quality in the State of São Paulo*. São Paulo: CETESB, **2022.**
